# Recent Progress in Electrochemical Nano-Biosensors for Detection of Pesticides and Mycotoxins in Foods

**DOI:** 10.3390/bios13010140

**Published:** 2023-01-14

**Authors:** Zhaoyuan Gong, Yueming Huang, Xianjing Hu, Jianye Zhang, Qilei Chen, Hubiao Chen

**Affiliations:** 1 Institute of Basic Research in Clinical Medicine, China Academy of Chinese Medical Sciences, Beijing 100700, China; 2 School of Chinese Medicine, Hong Kong Baptist University, Hong Kong 999077, China; 3 Guangdong Provincial Key Laboratory of Research and Development of Natural Drugs, School of Pharmacy, Guangdong Medical University, Dongguan 523808, China; 4 Guangzhou Municipal and Guangdong Provincial Key Laboratory of Molecular Target and Clinical Pharmacology, The NMPA and State Key Laboratory of Respiratory Disease, School of Pharmaceutical Sciences and the Fifth Affiliated Hospital, Guangzhou Medical University, Guangzhou 510000, China

**Keywords:** electrochemical biosensor, mycotoxins, pesticides, nanomaterials, gold nanoparticles, carbon nanotubes

## Abstract

Pesticide and mycotoxin residues in food are concerning as they are harmful to human health. Traditional methods, such as high-performance liquid chromatography (HPLC) for such detection lack sensitivity and operation convenience. Efficient, accurate detection approaches are needed. With the recent development of nanotechnology, electrochemical biosensors based on nanomaterials have shown solid ability to detect trace pesticides and mycotoxins quickly and accurately. In this review, English articles about electrochemical biosensors in the past 11 years (2011–2022) were collected from PubMed database, and various nanomaterials are discussed, including noble metal nanomaterials, magnetic metal nanoparticles, metal–organic frameworks, carbon nanotubes, as well as graphene and its derivatives. Three main roles of such nanomaterials in the detection process are summarized, including biomolecule immobilization, signal generation, and signal amplification. The detection targets involve two types of pesticides (organophosphorus and carbamate) and six types of mycotoxins (aflatoxin, deoxynivalenol, zearalenone, fumonisin, ochratoxin A, and patulin). Although significant achievements have been made in the evolution of electrochemical nano-biosensors, many challenges remain to be overcome.

## 1. Introduction

The widespread use of pesticides to control pests on fruits and vegetables has become a major issue in food safety. They improve crop yields while killing a variety of pests [1]. By chemical structure, the main categories of pesticides include organochlorines, organophosphorus, carbamates, chlorophenols, and synthetic pyrethroids [2,3]. Among them, the most toxic to human health are organophosphorus (OP) and carbamate (CB) pesticides, which inhibit acetylcholinesterase (AChE) and are responsible for a number of biochemical reactions [4] (Table 1).

Mycotoxins are natural harmful substances that come from certain types of molds. They belong to the Ascomycota family and consist of highly diverse organic structures characterized by functional groups containing various heteroatoms. Fungi are harmful to humans because of their abundant presence in the environment and food chain. Mycotoxins cause toxic reactions in higher vertebrates and other animals when they enter their bodies at low concentrations through natural routes. Most mycotoxins have unique chemical and thermal stability, making them difficult to remove during conventional food processing and disinfection [5,6]. Of the 300 known mycotoxins, six common mycotoxins, namely, aflatoxin (AF), deoxynivalenol (DON), zearalenone (ZEN), fumonisin (FM), ochratoxin A (OTA), and patulin (PAT), have had a long-term and sustained impact on global food safety issues [7]. Their hazards are assessed by the Joint Expert Committee on Food Additives (JECFA), a scientific advisory body to the Food and Agriculture Organization (FAO), and the World Health Organization (WHO) (Table 2).

Therefore, developing an efficient analytical procedure for determining pesticides and mycotoxins in various matrices is an important and urgent issue. In the past decades, the classic strategies to address this problem have mainly included (1) liquid chromatography coupled with fluorescence detection (LC-FLD) [8,9], diode-array detection (DAD) [10], or mass spectrometry detection (MS) [11,12], (2) gas chromatography (GC)-MS [13,14], and (3) thin-layer chromatography (TLC) [15]. They have been favored by testing organizations in the past because of their sensitivity and high accuracy. However, these methods usually involve tedious sample pretreatment, instrument operation, and human labor. These problems seriously hindered the real-time detection of pesticide residues and mycotoxins; therefore, great efforts have been made to explore alternative measures for detection in a highly selective, sensitive, and efficient manner [16]. In this context, biosensors can be a viable alternative to overcome the shortcomings of traditional analytical methods by simplifying or removing the sample preparation phase [17].

Biosensors are composed of receptors, transducers, and biorecognition substances that detect certain specific target molecules in the medium. Electrochemical biosensors based on biochemical interactions have high specificity and sensitivity, which offer simple, cost-effective, and mass-producible electrodes. They are expected to be used to develop small portable devices for the simultaneous analysis of multiple compounds [18]. Incorporating nanomaterials into devices can make the sample analysis more accurate, cost-effective, and efficient [19,20]. In the last decade, the use of nanomaterials in electrochemical biosensors has increased dramatically, achieving enhanced performance, improved selectivity, and sensitivity [21]. Magnetic nanoparticles (MNPs), metallic nanomaterials, carbon nanotubes (CNTs), graphene, and its derivatives are among the commonly used nanomaterials for detecting pesticides and mycotoxins.

This review summarizes recent advances in electrochemical biosensors combined with nanomaterials for detecting food pesticides and mycotoxins. The detection targets involve six categories of mycotoxins and two categories of pesticides. We herein investigate the role of nanomaterials in sample analysis, compare the sensors’ sensitivity and stability, and discuss challenges and possible solutions. It is expected that more advanced sensing technologies based on nanomaterials will be developed in the coming years, which will contribute to food safety and public health.

**Table 2:** The main hazards of six representative categories of mycotoxins to human health.

Mycotoxin	Fungal Sources	Health Hazards	IARC ^a^ Classification	Reference
Aflatoxins B1, B2, G1, G2	*Aspergillus flflavus* *A. parasiticus*	Acutely toxic, carcinogenic, immunosuppressive, reproductive toxicity	Group 1	[22]
Ochratoxin A	*A. ochraceus* *Penicillium verrucosum* *A. carbonarius*	Carcinogen, nephrotoxic	Group 2B	[23]
Fumonisins B1, B2	*Fusarium verticillioides* *F. proliferatum*	Acutely toxic, carcinogenic, immunosuppressive, hepatotoxic, and nephrotoxic	Group 2B	[24,25,26,27]
Zearalenone	*F. graminearum* *F. culmorum*	Reproductive toxicity and immunosuppressive	Group 3	[28,29]
Deoxynivalenol	*F. graminearum* *F. culmorum*	DON contamination of grains has been linked to human cases of fever, stomach pain, headache, vomiting, and diarrhea.	Group 3	[30,31,32]
Patulin	*P. expansum*	Immunotoxic, neurotoxic, hepatotoxic, and nephrotoxic	Group 3	[33,34,35]

^a^ IARC: International Agency of Research on Cancer.

## 2. Nano-Electrochemical Biosensors for Pesticides

### 2.1. Metal Nanomaterials

#### 2.1.1. Gold Nanoparticle (AuNPs)

Gold nanoparticles, negatively charged hydrophobic colloids, are among the earliest and most widely studied nanomaterials. Due to its high specific surface area, straightforward particle preparation, uniform particle size control, ability to increase catalyst size by silver deposition, and efficient surface modification by thiols or other bioligands [36,37,38]. Its unique biosensor properties and low toxicity make it a promising general-purpose platform for immobilizing antibodies (Ab), enzymes, aptamers (Apt), DNA, and other biomolecules [39]. The presence of AuNPs provides a suitable microenvironment for immobilizing biomolecules and reduces electron transfer resistance.

For the immobilization of Ab, Talan et al. developed a fluorine-doped tin-oxide electrode (FTO)-based electrochemical nanosensor for chlorpyrifos detection with AuNPs and Ab. The AuNPs were physically adsorbed on the surface of the FTO electrode to provide a homogeneous layer to obtain high conductivity. At the same time, AuNPs offered a platform to immobilize Ab through ionic or hydrophobic interactions. A specific antigen was then added for chlorpyrifos detection, resulting in significant current changes [40].

For the immobilization of enzymes, Song et al. created a carbamate pesticide sensing interface based on a citrate-capped AuNPs/(3-mercaptopropyl)-trimethoxysilane (MPS)/gold electrode (AuE). The negatively charged AuNPs/MPS/AuE interface repelled the equally charged [Fe(CN)_6_]^3−^/^4−^, producing a negative response when ferricyanide ([Fe(CN)_6_]^3−^/^4−^) was used as the redox probe. AChE catalyzed the hydrolysis of acetylthiocholine iodide (ATCI) to positively charged thiocholine. Thiocholine can then be fixed on AuNPs by replacing citrate with an Au–S bond, turning the negatively charged surface into a positively charged surface and attracting the negatively charged [Fe(CN)_6_]^3−^/^4−^, producing a corresponding positive response [41]. Since carbamate pesticides can inhibit AChE activity, the sensing interface exhibited a satisfactory sensitivity. In another report, AuNPs were electrodeposited on screen-printed carbon electrodes (SPCE) to covalently immobilize AChE via the Au–S bond. After the formation of the sensor, ATCI was used as the substrate to reflect the activity of AChE. The fabricated transducer showed high potential in detecting OP pesticides [42].

Due to the unique optical properties of AuNPs, the localized surface plasmon resonance (LSPR) technology based on sensors composed of AuNPs has been gradually applied to detect biomolecules. Li et al. developed a sensor probe functionalized with graphene oxide (GO), AuNPs, molybdenum disulfide nanoparticles (MoS_2_-NPs), and creatininase enzyme. The concentration of creatinine was determined by optical fiber LSPR [43]. Zhu et al. immobilized AuNPs and GO on the bare probe. Then ascorbic acid oxidase was functionalized. The great potential of ascorbic acid (AA) sensor in practical application of daily diagnosis was developed [44]. Singh et al. immobilized a layer of AuNP between the GO layer and the fiber surface to form a tapered optical fiber sensor probe. The selectivity of the sensor to uric acid (UA) was improved by functionalizing the nanomaterial-coated fiber with uricase enzyme [45]. Other biological molecules, such as AchE and heparin, can also be detected using AuNPs. Based on the competitive host guest interaction between sulfonated calix [6] arene (p-SC6A)-terminated AuNPs and rhodamine B (RhB)/acetylthiocholine, Lv et al. developed a fluorescent and colorimetric dual channel probe for rapid detection of AChE with high sensitivity and selectivity [46]. Unser et al. proposed a functional plasma protein scaffold by integrating collagen fibers with negatively charged citrate-capped gold nanoparticles. With the multiple functions of collagen, protein nanoparticle scaffold is used to detect its specific interaction with glucose and heparin through plasma coupling [47].

#### 2.1.2. Sliver Nanomaterials (AgNMs)

AgNMs are also commonly used in the manufacture of biosensors. Among all metals, silver (Ag) has the highest electrical and thermal conductivity and reflectivity. It is almost completely harmless to the human body. Representative Ag-based nanosensors include Ag nanoparticles (AgNPs), Ag nanorods (AgNRs), and Ag nanowires (AgNWs) [48].

For AChE immobilization, Lang et al. compared the performance of sensors composed of AuNRs and Au–Ag heterogeneous nanorods (AuAgNRs). Compared with AuAgNRs, AuNRs exhibited better catalytic performance and lower detection limits. The results showed that, when it comes to AChE immobilization, the silver surface did not show higher catalytic sites than gold [49].

However, AgNMs are suitable for butylcholinesterase (BChE) immobilization. The two cholinesterases have some differences in substrate binding, catalytic mechanism, and sensitivity to enzymes [50]. In order to assess the paraoxon content in multiple matrices, Turan et al. described a BChE amperometric biosensor combining AgNWs with a conducting polymer. First, a polymer film was created by electrochemical polymerization of the compound 5,6-bis(octyloxy)-4,7-bis(thiopheno[3][3,2-b]thiophene-2-yl)benzo[c][1,2,5]oxadiazole (TTBO) over the surface of a graphite electrode. Subsequently, BChE was cross-linked on the modified electrode surface via FA crosslinking. The results showed that biomolecules can maintain their activity under the best experimental conditions [51].

#### 2.1.3. Magnetic Nanomaterials

Magnetic nanoparticles (MNPs) are nanoscale particles that are generally composed of iron (Fe), cobalt (Co), nickel (Ni), and other metal oxides. A class of nanomaterial with distinctive physicochemical characteristics and a staggering array of uses is MNPs. MNPs can simplify complicated traditional experimental procedures and shorten the experimental time because of their distinct physical and chemical properties. Especially since iron oxides (Fe_3_O_4_) are biocompatible and facilitate easy preparation and functionalization, they are commonly used in biological analysis. MNPs are usually used as electrode modifiers in the detection technology integration of pesticide residue determination. When used as electrode modifiers, MNPs can significantly enhance the electron transfer between analytes and electrodes due to their very high charge transfer capability.

**Figure 1:**
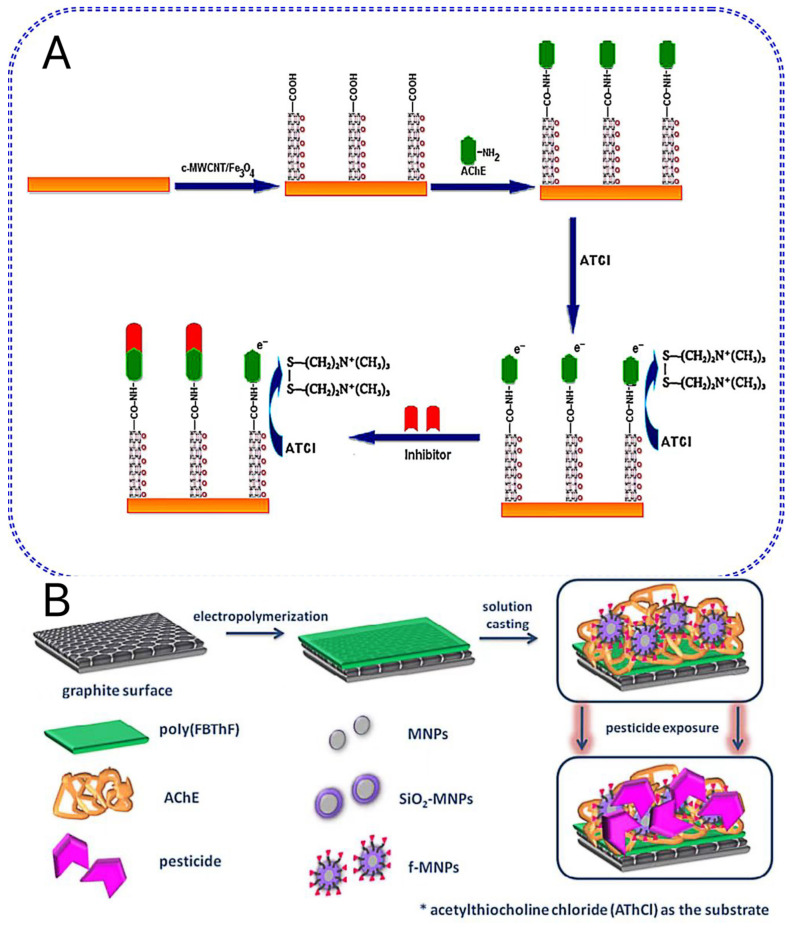
Schematic illustration of the stepwise amperometric biosensor fabrication process and immobilized acetylcholinesterase inhibition in pesticide solution (**A**). Reproduced with permission from Chauhan and Pundir [52] Anal. Chim.Acta; published by the Elsevier B.V., 2011. Schematic representation of the biosensor (**B**). Reproduced with permission from Dzudzevic Cancar et al. [55] ACS Appl. Mater Interfaces; published by the American Chemical Society 2016.

Unique properties of magnetic Fe_3_O_4_ nanoparticles include their huge surface area, excellent chemical stability, and low toxicity. Therefore, biomolecules (such as AChE) can be fixed on iron oxide nanoparticles (Fe_3_O_4_NP) to build a sensor for detecting OP. Fe_3_O_4_NP and carboxylated multi-walled carbon nanotubes (c-MWCNT) were utilized by Chauhan and Pundir to modify AuE. Following the decoration of Fe_3_O_4_ nanoparticles onto the surface of c-MWCNT, carboxyl groups (-COOH) that were present on the external surface of MWCNTs were free to bond with amino groups (-NH_2_) on the surface of AChE. The sensor had good sensitivity, reusability, and durability, making it appropriate for the trace detection of OP pesticide residues in milk and water [52] (Figure 1A).

The aggregation of MNPs can be prevented by coating carbon materials, which also offers large-area support for potential modification. The properties of the obtained materials can be further enhanced by combining MNPs with large-area carriers (such as carbon spheres) [53]. AChE sensors were created in a study using mesoporous hollow carbon spheres (MHCS) and magnetic mesoporous hollow carbon spheres with core–shell structures (Fe_3_O_4_/MHCS). Glutaraldehyde (GA) was used as a cross-linker to immobilize AChE into nanocomposites without destroying the activity of AChE. The created sensor demonstrated good stability, particularly with the addition of Fe_3_O_4_NP, which improves the sensor’s stability [54].

Silica coating also can prevent the aggregation of MNPs and improve biocompatibility. In addition, it provides a suitable platform for further surface modification through various functional groups. Dzudzevic Cancar et al. synthesized core–shell MNPs sequentially modified with silica and -COOH. The MNPs’ silica inner shell and carboxyl outer skeleton maintain the nanoparticles in solution and offer locations for biomolecules to connect covalently. The carboxyl group of MNPs can form amide bonds with various enzymes, maintaining the enzyme’s catalytic activity and random orientation. In this regard, a graphite electrode was reported with its surface electrochemically polymerized with 4,7-bis (furan-2-yl) benzo [c] [1,2,5] thiadiazole (FBThF); the polymer surface was then modified with the -COOH and -SiO_2_-functionalized magnetic nanoparticle (f-MNP) and AChE to form a biosensor for OP from tap water samples [55] (Figure 1B).

#### 2.1.4. Metal–Organic Framework

A crystalline material called a metal–organic framework (MOF) is composed of inorganic metal centers (metal ions or clusters) joined by organic ligands. It offers unparalleled tunability, a large surface area, high porosity, excellent catalytic activity, and a wealth of active sites [56]. It can be used as a highly selective platform for the development of sensors related to electrochemical detection. Numerous MOF materials have been applied to construct electrochemical sensors, including copper-based (Cu-MOF) [57], zirconium-based (Zr-MOF), nickel-based (Ni-MOF) [58], iron-based (Fe-MOF), and cobalt-based MOF (Co-MOF). In order to further endow more functions and improve its performance, it is an effective strategy to modify MOF by introducing heteroatoms, functional groups, and metal ions.

Deep et al. described the assembly of nano-MOF (NMOF) on a parathion sensing electrode. The indium tin oxide (ITO) electrode slide was modified by 2-aminobenzylamine (2-ABA) and sequentially dipped into the organic linker 2-aminoterephthalic acid and the metal ion “Cd^2+^” solutions. A derivative of aniline, 2-ABA, was electrochemically deposited on the ITO surface, freeing –NH_2_ moieties to react with the –COOH groups of the organic linker, on which the metal center (Cd) subsequently assembled to grow a rod-like NMOF structure. The available –COOH groups immobilized anti-parathion antibody on the NMOF sensor, resulting in sensitive and selective analysis of parathion in a rice sample [59].

#### 2.1.5. Other Metal/Metal Oxide Nanoparticles

As an analog of graphene, two-dimensional (2D) transition metal dichalcogenides (TMDs), include MoS_2_, WS_2_, and MoSe_2_. Through the interaction of van der Waals forces, these materials are composed of transition metal atomic layers sandwiched by two chalcogenide atomic layers and further stacked into multiple layers. Molybdenum disulfide (MoS_2_) is a graphene analog stacked by covalently bonded S-Mo-S. In order to solve the problem that the AuNPs coating on the electrode surface easily fell off in the measurement process, monolayer MoS_2_ nanosheets were reported in advanced biosensors to secure AuNPs by forming solid Au–S bonds [60]. For effective paraoxon detection, Jia et al. assembled an AuNPs/MoS_2_/rGO/polyimide (PI) composite electrode. PI is a polymer with good film-forming properties. A wide potential range, high mechanical properties, flexibility, and temperature tolerance are all produced by the ideal interaction between rGO and PI [61,62]. Researchers further modified the rGO/PI electrode with MoS_2_ and AuNPs, where the MoS_2_ monolayer can reduce the AuNPs. Finally, the formed AuNPs/MoS_2_/rGO/PI flexible film is used to immobilize AchE for paraoxon biosensing [63]. As another example, Zhao et al. utilized metallic MoS_2_ nanosheets as the electrochemical platform to develop a disposable enzyme-based pesticide biosensor. In order to improve the performance of the sensor, AuNPs were electrodeposited in situ on the SPE surface. The results showed that the exfoliated thin metal MoS_2_ nanosheet had suitable electron transfer properties and good immobilization effect on AchE. With ATCI as the substrate, the biosensor has satisfactory electrocatalytic activity for the electrochemical oxidation of thiocholine [64]. Representative examples of such biosensors are listed in Table 3.

### 2.2. Carbon-Based Nanomaterials

#### 2.2.1. Carbon Nanotubes

The cylindrical nanostructures that build carbon nanotubes (CNTs) offer great physical properties, such as low density, significant porosity, and excellent conductivity. To create CNTs, single-layer graphite sheets are rolled into tubes, or multiple-layer graphite sheets are rolled into compressed cylinders [65]. As a promising material, they are often used to be incorporated into immunosensors in different ways. They are often coated on carbon electrodes to improve the selectivity and sensitivity of small molecule electrochemical assays. At the same time, the electrode surface area and adsorption density can be increased.

SWCNT or MWCNT needs further modification to immobilize biological molecules. In one study, MWCNTs were deposited with reducing gold salts and then treated with acid, in order to introduce hydrophilic functional groups such as -OH and -COOH. Functional groups provided anchor sites for metal nanoparticles and biological enzymes. The outer surface of MWCNT was modified by AuNPs, which is expected to improve the immobilization of enzymes and the electron transfer rate between reaction sites and electrodes [66]. In a subsequent study, SWCNT and MWCNT were used in combination. Carboxylated SWCNT surfaces were used to immobilize AchE, while MWCNTs were used to enhance the electrocatalytic activity of the electrodes. In order to create the working electrode’s core electrode, a gold wire was coated with AuNPs and MWCNT paste. AchE was immobilized on carboxylated SWCNT, then pasted onto the electrode core, and then coated with a Nafion layer which acted as binder to prevent the enzyme from leaching from the electrode. Excellent stability and reusability were displayed by this biosensor [67]. Introducing heteroatoms into SWCNTs can change their physical and chemical properties and improve their conductivity. Nitrogen is the ideal dopant for SWCNTs since its atomic radius is similar to that of carbon. As a result, C–N bonds make it simple to access SWCNTs [68].

To overcome the disorder and aggregation problem of SWCNT, an advanced version, vertical nitrogen-doped single-walled carbon nanotubes (VNSWCNTs) were developed. In one practice, VNSWCNTs were produced by spontaneous chemisorption of thiol-functionalized SWCNTs, where AuNPs were electroless plated on the surface. Subsequently, AchE was fixed on the AuNPs via Au–S bonds. The constructed AchE-immobilized biosensor was reported with excellent detection of methanol in cabbage water [69].

#### 2.2.2. Graphene and Its Derivatives

Graphene is a hexagonal network of covalently bonded sp^2^ hybrid carbon atoms. It possesses unique physical and chemical properties, especially high conductivity, a sizable specific surface area, low toxicity, and high electron mobility [70,71,72,73]. In addition, GO with a variety of oxygen-containing chemical groups can bind biomolecules easily without additional activation, and it has excellent hydrophilicity and can be easily synthesized in the laboratory at very low cost [74].

For AchE immobilization, graphene nanocomposites perform better performance than pure graphene in the manufacturing of biosensors. A biosensor with AchE immobilization on CdS-decorated graphene (CdS-G) nanocomposite was reported by Wang et al. The nanocomposites had good electron transfer channels, and the immobilized AchE had high enzyme activity and affinity for ATCI. The AchE’s active site can be more easily contacted when OP was in contact with CdS-G nanocomposites, which reduced the inhibition time [75].

**Figure 2:**
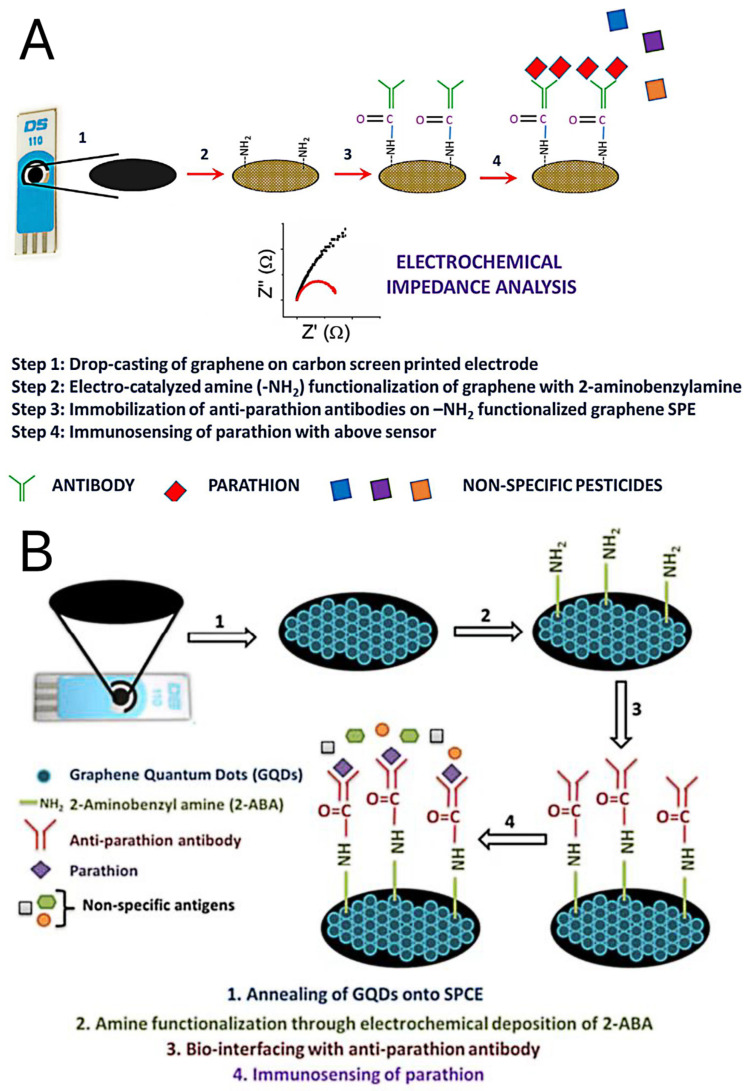
Schematic of the graphene-based, screen-printed immunosensor for parathion (**A**). Reproduced with permission from Mehta et al. [76], Biosens. Bioelectron.; published by 2016 Elsevier B.V. Schematic presentation of the GQDs-modified screen printed immunosensor for parathion (**B**). Reproduced with permission from Mehta et al. [77], Anal. Biochem.; published by 2017 Elsevier Inc. All.

For Ab immobilization, Mehta et al. used the graphene sheets (GS) to modify the screen-printed carbon electrodes (SPCE). The electrode surface was electrochemically treated with 2-ABA to generate active -NH_2_ functional group. The amine-functionalized graphene electrodes were biologically linked to the ferrocene (Fc) region of anti-parathion Ab through amide bonds (-CO–NH-). The biological conjugation process can be carried out directly without cross-linking agents or any other cumbersome chemicals [76] (Figure 2A). A follow-up study replaced GS with amine-functionalized graphene quantum dots (GQD) to form electrochemical biosensors. It was simple to decorate the GQD with more homogeneity on the screen-printed surface, which improved the accuracy and precision of the sensor control. Additionally, the presence of functional groups in situ on the surface of GQD makes it easier for the 2-ABA moiety to firmly assemble and promote the amine function that modifies the anti-parathion antibody on the sensor. Compared with the previous model, the sensor composed of GQD provides a lower detection limit and a broader analysis range [77] (Figure 2B; Table 4).

### 2.3. Aptamer-Based Nanoparticles

The SELEX technique, a rigorous in vitro chemical selection method, is used to create Apts, which are short nucleotide sequences of either single-stranded ribonucleic acids (ssRNAs) or single-stranded deoxyribonucleic acids (ssDNAs) [78]. Recently, efforts have been made to develop aptasensors for detecting pesticides.

Hong et al. identified four unique malathion-specific ssDNA Apts. Among them, two Apts showed high affinity to thioflavin T (ThT) and produced strong fluorescent signals. On such basis, two independent sensing strategies using fluorescent labeling and ThT displacement were designed. When malathion is combined with aptamer, it can be directly detected by fluorescence. When malathion with higher concentration is added to the sample, the fluorescence intensity decreases proportionally, indicating that malathion directly replaces the ThT dye in the determination. The selected aptamers formed a G4-tetraploid-like (G4Q) structure, which facilitated the development of a label-free assay with a detection limit of 2.01 ppb [79].

In addition, Apts can also be immobilized onto nanomaterials to form sensors for the detection of pesticides. In one study, thiol-tethered DNA-Apt-captured probes were immobilized on gold nanoparticles/polyaniline composite membrane-modified electrodes (AuNPs–PANI). Profenofos solutions containing a fixed amount of biotinylated DNA-Apt were dropped onto the aptasensor, with practically sufficient detection sensitivity. The hybridization reaction was measured using a streptavidin alkaline phosphatase conjugate that catalyzes the hydrolysis of 1-naphthyl phosphate. The 1-naphtholase product was detected by competitive binding [80]. In another study, AuNPs were electrodeposited on the surface of 2D Mo_2_C/Mo_2_N composite-modified electrode to connect with ferrocene (Fc) probe through Au–S bond. The Fc probe can hybridize with the Apt-probe to form a double-chain structure. The addition of chlorpyrifos (CPF) melted the double-chain, and the Fc probe was close to the electrode surface, which led to the amplification of the electrochemical response. The aptasensor was used to detect CPF in apple and cabbage samples with satisfactory recovery [81]. Xu et al. electrodeposited polydopamine-AuNPs (PDA-AuNPs) on the electrode surface and added exonuclease I (ExoI) to form a dual signal aptamer sensor for the detection of malathion. The 5‘and 3’ hairpin probes labeled with Fc and sulfhydryl groups respectively were fixed on the electrode by an Au–S bond and hybridized with the 5‘carboxylated Apt. Sulfur (Tn) with amino group is modified by 1-ethyl-3-(3-dimethylaminopropyl) carbodiimide/N-hydroxysuccinimide (EDC/NHS) as the second signal of Apt 5‘. After malathion was added, the aptamer malathion complex was formed by aptamer hybridization, which led to the melting of double chains and the weakening of Tn electrochemical signal. The sensor has high sensitivity and good selectivity, and was successfully applied to the practical detection of vegetable samples [82] (Table 5).

## 3. Nano-Electrochemical Biosensor for Mycotoxins

### 3.1. Metal Nanomaterials

#### 3.1.1. Gold Nanoparticle (AuNPs)

There have been multiple reports on AuNPs-based mycotoxin biosensors with Ab immobilization. For example, it is reported that horseradish peroxidase (HRP) and anti-AFB1 Ab can self-assemble onto AuNP-functionalized biological cognitive surfaces, forming a micro-comb electrode. When AFB1 in the sample solution reacted with the anti-AFB1, a direct electrical communication barrier was be formed between the immobilized HRP and the electrode surface [83]. In another study, Nafion 117 was used as a binder to form a film of AuNPs-dotted 4-nitrophenylazo-functionalised graphene (AuNPs/G/PhNO_2_) composite onto an electrode. With [Ru(bpy)_3_]^2+^ as the redox probe, the decorated electrode can then immobilize deoxynivalenol antibody for wheat, roasted coffee, and corn analysis [84].

For Apt immobilization, Jalalian et al. created an electrochemical aptasensor for the detection of AFM1. The hairpin-shaped structure of AFM1 Apt specifically binds to the thiol-modified complementary strand of the Apt(CS), and is immobilized on the electrode through AuNPs. High sensitivity and selectivity for the detection of AFM1 were made possible by the conformational change in the Apt’s hairpin-shaped structure in the presence and absence of both negatively charged AuNPs and AFM1. The Apt’s hairpin structure was maintained even without the AFM1. So, a weak peak current was obtained. The hairpin shape of the Apt might be destroyed by the inclusion of AFM1 [85]. The Apt chain is fixed to the composite of AuNPs and covalent organic frameworks (COFs) through Au–SH bond, with H_2_O_2_ as the redox probe. As the concentration of ZEN in the solution rises, it specifically binds to ZEN Apt, prevents electron transfer, and limits the catalytic current of AuNPs/COF for the reduction of hydrogen peroxide. By monitoring the declining catalytic current, the ZEN toxin was quantitatively detected [86].

Subak et al. electrochemically modified the electrode using PANI and AuNPs (AuNPs–PANI) as a scaffold for immobilizing the thiol-Apt. The binding region of the oligonucleotide sequence can be found by docking research, and the preferred orientation of DON can be determined. The biotinylated oligonucleotide sequence CS to the Apt was selected for competitive format. The binding region of oligonucleotide sequence can be found by docking study, and the preferred orientation of DON can be determined. A solution containing increasing concentrations of DON and a fixed amount of CS was dropped onto the surface of an aptasensor and detected by differential pulse voltammetry (DPV) [87].

**Figure 3:**
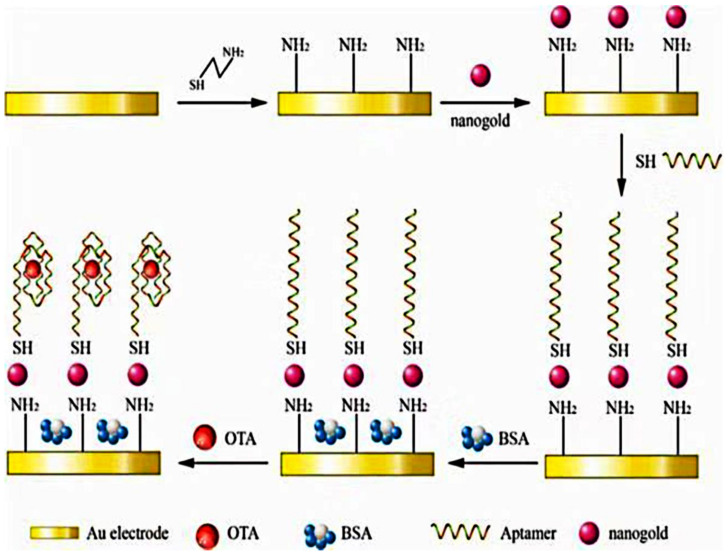
Schematic presentation of the fabrication steps and principle of the label-free impedimetric aptasensor. Reproduced with permission from Nan et al. [90], Toxins (Basel); published by 2019 MDPI.

Layer-by-layer (LBL) self-assembly is one of the simplest methods to immobilize biomolecules on sensors [88]. On such basis, the Apt can be easily fixed efficiently. Yang et al. reported the synthesizing process of an Apt-based OTA biosensor. First, the sulfhydryl group of cysteamine (CA) molecule forms a chemical bond with a bare AuE to form a self-assembled monolayer film. After that, the AuNPs were fixed on the surface of CA-modified electrode through the electrostatic interaction between the negatively charged citrate around them and the positively charged amino group of CA. AuNPs with a larger surface area than bare AuE can immobilize more Apt. The thiolated Apt binds to AuNPs by the Au–S bond. The self-assembled monolayer of the Apt is negatively charged because the deconvolution of the Apt leads to alkali exposure [89], which directly leads to electrostatic repulsion between the Apt and the redox probe. After bovine serum albumin (BSA) modification, the label-free impedimetric aptasensor was prepared and used for the determination of OTA (Figure 3) [90].

#### 3.1.2. Gold Nanorods (AuNRs)

**Figure 4:**
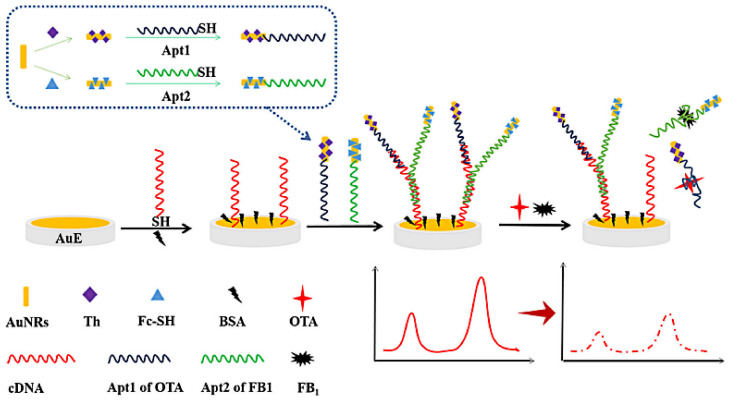
Schematic representation of OTA and FB1 detection based on Apt2-AuNRs-Fc/Apt1-AuNRs-Th/cDNA/AuE. Reproduced with permission from Wei et al. [93], Microchim. Acta; published by 2020 Springer Nature Switzerland AG.

AuNRs are more sensitive to the dielectric environment and significantly more sensitive to quantify biological molecules than Au spherical nanoparticles due to their higher absorption and unique longitudinal localized surface plasmon resonances [91,92]. Wei et al. hybridized OTA Apt and FB1Apt with their CS simultaneously to form a unique Y-shaped DNA structure. Then they were immobilized on AuNRs-modified electrodes, and the composed sensor can simultaneously detect OTA and FB1. The designed experimental principle is shown in Figure 4. AuNRs had the advantage of high affinity and high electric transfer ability to mercaptan-modified molecules. It was used to immobilize electrochemical probes including Thi and SH-Fc and SH-Apt to form amplified signal elements and molecular recognition elements [93].

#### 3.1.3. Sliver Nanomaterials (AgNMs)

For immobilization of Apt, Karimi et al. immobilized Apt on citrate-capped AgNPs via their amino groups. The transient current generated upon individual impact of Apt-coupled AgNPs with carbon fiber microelectrodes (CFME) was detected by nano-impact electrochemistry. For real-time quantitative detection of the OTA with high sensitivity, the change in current response brought on by the collision of conjugated Apt with AgNPs can be employed [94].

#### 3.1.4. Bimetallic Nanomaterials

Bimetallic nanomaterials change the electronic structure by adding a second metal element to further adjust the adsorption strength of reactive molecules. It can change in chemical and physical properties, such as catalytic activity and chemical selectivity [95,96], through interactions between the two components.

Palladium nanoparticles (PdNPs) and platinum nanoparticles (PtNPs) can be used to amplify electrical signals. Pd-PtNPs have stronger catalytic activity for H_2_O_2_ than PdNPs or PtNPs [97]. Ji et al. created 3D sakura-shaped nanostructures using metal organic coordination polymers (MOCPs) fabricated of L-Glu and Cu^2+^. MOCP improved the sensor signal enhancement capability by loading more metal ions. In order to label CS, which can catalyze the reduction of H_2_O_2_, amplify the detection signal, and link Apt, MOCP/Pd-PtNPs nanocomposites were utilized. Then the conductive polymer polyaniline (PANI) was used to modify AuNPs, and spherical Au-PANI-Au nanohybrid was prepared to modify the electrode. It can assemble a large number of Apt-labeled amino groups due to the strong Au–NH_2_ bond [98], thus further improving the sensitivity of the aptasensor [99]. In addition, gold-platinum alloy nanoparticles (AuPtNPs) are one of the most useful bimetallic nanoparticles. Due to the highly synergistic effect between gold and platinum, they have an excellent catalytic effect [100].

Based on the oriented immobilization of a MAb specific for ZENs, Liu et al. developed a label-free immunosensor. First, MWCNTs were functionalized by polyethylenimine (PEI) to modify an electrode, which was used to support nanoparticles and improve the electronic conductivity and chemical stability of the electrode. AuPtNPs were electro-deposited, which increased the surface area for capturing a large number of antibodies and enhances the electrochemical performance. Staphylococcal protein A (SPA) was immobilized in AuPtNPs. Four Fc binding domains in SPA can bind to the antibody’s Fc region with specificity and release the antigen binding site [101,102]. The assay offers a sensitive and practical technique for the detection of such mycotoxins because it is extremely repeatable and selective [103].

#### 3.1.5. Magnetic Nanomaterials

He and Yan proposed an electrochemical aptasensor by employing hollow cubic gold-platinum nanoframes (hcPtAuNFs)-functionalized PEI-rGO as the label material. This label material has good conductivity and large specific surface area, which can increase the load of thionine (Thi) and CS1. Then Fe_3_O_4_ nanorods (Fe3O4NRs) and rGO were used to build a platform to modify on gold electrodes to load AuNPs. It can not only effectively improve the load capacity of CS2, but also catalyze Thi and CS2. Upon the specific recognition of Apt to ZEN, CS2 were loaded on AuNPs captured CS1 fixed label material through hybridization reaction. This aptasensor had good specificity, stability, and repeatability [104].

#### 3.1.6. Metal–Organic Framework

Wen et al. created a simple electrochemical aptasensor based on assembling multifunctional nitrogen-doped copper metal–organic backbone (N-Cu-MOF) nanomaterials and Apt. N-Cu-MOF, with a large specific surface area and good conductivity, can be used as the best electrical signal probe, but also as an effective support matrix for stabilizing the Apt through the interaction of NH_2_ and copper. When the electrode was assembled with Apt, the electrical signal of N-Cu-MOF disappeared because the -NH_2_ in Apt is complexed with copper. This means that the electrochemical activity of copper-based nanomaterials is restored after Apt is removed from the N-Cu-MOF-modified electrode [105].

Due to its ideal nanoscale size and superior packaging efficiency, Zr-MOF can offer the accessibility needed by methylene blue (MB). He and Dong used Zr-MOF-labeled oligonucleotides load with MB as signal probes. The sensor was constructed by using Au-Pt core–shell nanorods, iron-based metal organic framework (Fe-MOF) and PEI-rGO composite as modified electrodes, and DNA walking machine as electrocatalytic enhancer. A common form of Fe-MOF as the core metal and highly catalytic activity for dye molecules, such as MB, Thi, etc., were cited [106,107]. DNA walking strategy can trigger hundreds of signal molecules in response to a single binding event with high specificity. The sensor triggered nicking endonuclease (Nb. BbvCI) by adding PAT to power the DNA walking machine, which can effectively release Zr-MOFs/MB, this caused a significant change in the MB signal. PAT concentration was connected to the change in the MB signal [108].

**Figure 5:**
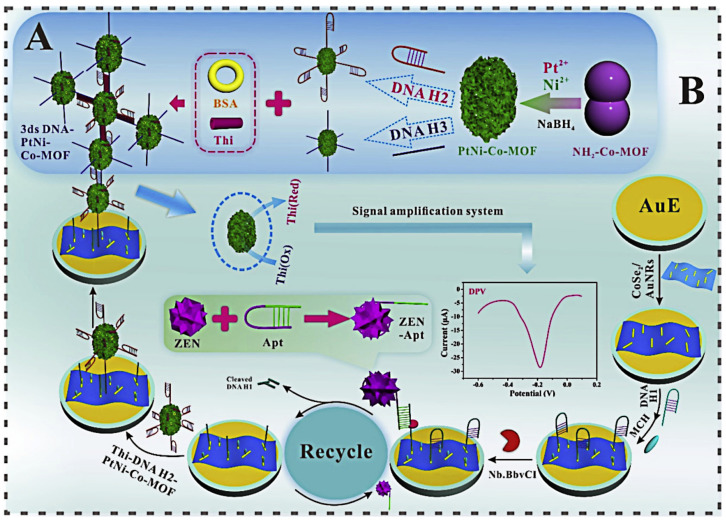
Illustration of (**A**) the fabricated Thi labeled 3ds DNA-PtNi@Co-MOF networks and (**B**) the fabricated aptasensor for ZEN detection. Reproduced with permission from He and Yan [110]. Sensors Actuators B. Chem.; published by 2019 Elsevier B.V.

He and Yan used Thi as a signal tags, PtNi nanoclusters (PtNi) and Co-MOFs were used to synthesize a signal amplifier. PtNi can immobilize DNA through Pt-N. At the same time, PtNi can also form a three-dimensional structure DNA-PtNi/Co-MOF through the Pt–S bond and Thi and has a synergistic catalytic effect on Thi with Co-MOF. AuNRs with high specific surface area and fast electron transfer capability were selected to modify the electrode and immobilize biological molecules with CoSe_2_ compounds [109]. The designed experimental principle is shown in Figure 5. The synthesized aptasensor has good stability and specificity, and can detect ZEN in corn samples [110].

#### 3.1.7. Other Metal/Metal Oxide Nanoparticles

**Figure 6:**
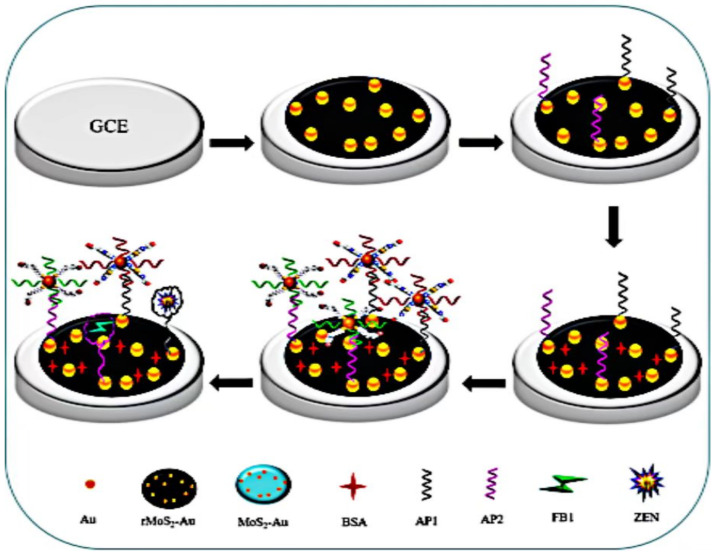
Schematic illustration of the preparation of a dual-target electrochemical aptasensor for multiplex detection of mycotoxins. Reproduced with permission from Han et al. [112], Biosens. Bioelectron.; published by 2019 Elsevier B.V.

MoS_2_ can form reduced MoS_2_ (rMoS_2_) through electrochemical reduction, which may further improve conductivity, electron transfer rate, and electrochemical activity and help to assemble and firmly couple to a large number of different Apt [111]. Han Z et al. used Co reduced molybdenum disulfide nanosheets and AuNPs (rMoS_2_-AuNPs) as an efficient platform to modify electrodes through Au–SH bond. By combining AuNPs with the Apt CS via the strong Au–SH and Au–NH_2_ bonds in an environmentally friendly way, the signals were further amplified. In addition, Thi and 6-(Ferrocenyl) hexanethiol (FC6S) were selected as probes. The designed experimental principle is shown in Figure 6. Actual maize samples were used to test the aptasensor’s efficacy, and satisfactory recoveries were made [112].

Zinc oxide nanorods (ZnONRs) is a versatile semiconductor material with biocompatibility, large specific surface area, relative chemical stability in a physiological environment, and electrochemical activity [113,114]. Then, ZnONRs were modified with chitosan. Chitosan contains wealthy amino groups. It can attach ZnO to the surface of AuE, and provide active sites for further immobilization of AuNPs [115]. The aptasensor was improved by the electrodeposition of AuNPs. Subsequently, thiol-modified Apt were self-assembled on AuNPs that were electrodeposited on the surface of the modified AuE. Under optimal conditions, the aptasensor shows good detection performance [116]. Representative samples of such metal-based biosensors are shown in Table 6.

### 3.2. Carbon-Based Nanomaterials

#### 3.2.1. Carbon Nanotubes

For the immobilization of enzymes, Li et al. created an amperometric biosensor based on oxidase. Aflatoxin oxidase (AFO) has selective oxidation activity to AFB1, which was embedded in a platinum electrode modified by multi-walled carbon nanotubes (MWCNTs) in a sol-gel. AFO is used to catalyze the oxidation of AFB1, release hydrogen peroxide, and generate electrical signals. The covalent bond between AFO and MWCNT maintains the enzyme activity. The ability of CNT to promote hydrogen peroxide electron transfer reaction shows that amperometric biosensors based on oxidase have broad prospects. A biocompatible microenvironment was created by the silica sol-gel surrounding the enzyme molecule, stabilizing its biological activity and preventing the enzyme from leaking out of the interface [117,118]. This biosensor can be shrunk, which adds to its benefits of having no label and makes it a portable screening equipment that is also affordable and has reusable electrodes [119].

**Figure 7:**
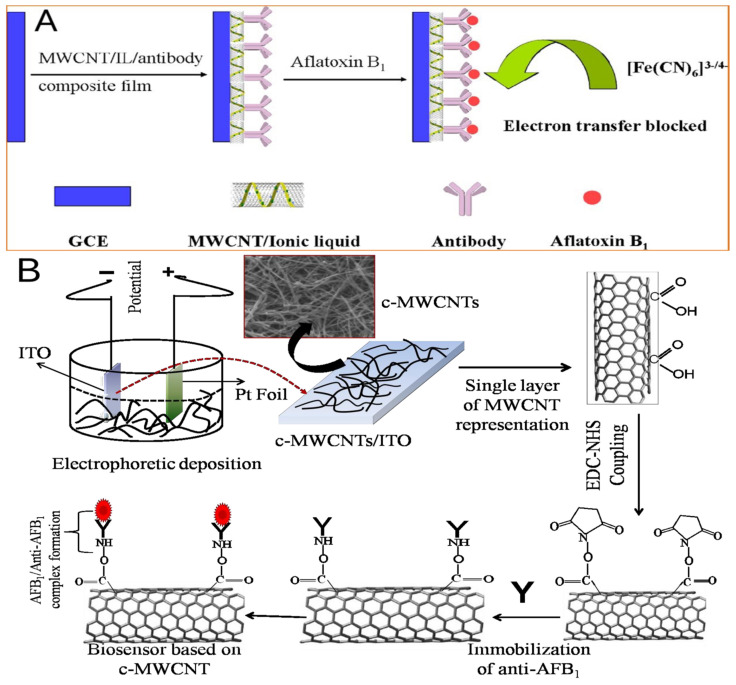
Schematic representation of c-MWCNTs-based biosensor for AFB1 detection proposed in (**A**). Reproduced with permission from Singh et al. [120], Sensors Actuators, B Chem.; published by 2013 Elsevier B.V. Schematic representation of the fabrication of the immunosensor proposed in (**B**). Reproduced with permission from Yu et al. [123], Food Chem.; published by 2014 Elsevier Ltd.83.

For the immobilization of Ab, Singh et al. used carboxylated multi-walled carbon nanotubes (c-MWCNT) to form covalently linked aminated antibodies through strong amide bonds (CO–NH) to establish the stability of the biosensor. C-MWCNT was deposited on indium tin oxide (ITO) substrate and used for immobilization of monoclonal anti AFB1 (anti-AFB1), and bovine serum albumin (BSA) solution was used to block the nonspecific active site of the electrode. The biosensor showed good reproducibility and stability within 45 days (show as Figure 7) [120].

Yu L. et al. used carboxylated MWCNTs, ionic liquids (ILs) and anti-AFB1 were immobilized on the traditional three electrode system. AFB1 was determined by electrochemical impedance spectroscopy (EIS). The MWCNTs in the ILs have a 3D network structure formed of significantly smaller, less tangled bundles that are physically cross-linked through cation-π and π-π interactions between imidazolium cations and CNTs [121]. For the immobilization of Ab and the transfer of small molecule hapten, the 3D network offers countless conductive microcavities [122]. The sensor detects AFB1 extracted from olive oil to test this method (Figure 7) [123].

In another study, MWCNT was modified with poly (2,5-dimethoxyaniline) (PDMA), and then PDMA-MWCNT was electrodeposited on the surface of glassy carbon electrodes (GCE). BSA mixture of Ab was dripped onto the composite polymer-modified GCE. After adding FB1, it was observed that the Ab layer and Ab-antigen interaction were non-conductive, so the electron transfer process of the developed immunosensor was inhibited [124]. SWCNTs/chitosan nanocomposites have good conductivity and can greatly enhance the electrochemical signal. Chitosan can enhance the dispersive property of SWCNTs [125,126].

Yang et al. established sensor was based on the indirect competitive binding between free FB1 and FB1-BSA and a fixed amount of anti-FB1 Ab, and the latter was bound to the covalently functionalized nanotube/chitosan immobilized on the GCE. In the form of the competition, more antigens were used for competitive recognition of anti FB1, and the upper limit of detection range was expanded. Finally, Ab conjugates had high catalytic activity for the enzyme substrate, a-naphthyl phosphate (a-NP), to obtain electrochemical signals [127].

For the immobilization of Apt, Abnous et al. used SWCNTs as electrochemical signal amplifiers, cross-linked to AuE by CS. Apt-containing modified AuE was used as a sensing ligand, and MB was used as a redox indicator to jointly construct an aptasensor. The duplex is broken down if OTA is introduced, and the MB and SWCNTs are liberated from the gold electrode’s surface. As a result, the electrochemical signal is weakened [128]. PEI is a polycationic polymer capable of interacting with the carboxylate groups present in the proteins [129]. It can improve the water solubility of nanocomposites and successfully prevent MWCNT aggregation and entanglement, increasing the effective surface area of MWCNTs. A study used MWCNT/PEI dispersion to deposit on the electrode surface. The modified electrode was immersed in AuNPs solution, and AuNPs were adsorbed on the amine group in the polymer. The aptasensor was prepared by combining Apt with AuNPs fixed on MWCNTs/PEI. Then ZEN was labeled with HRP to construct HRP/H_2_O_2_ system. The determination of ZEN was measured by H_2_O_2_ reduction current through amperometry, while HRP coupled with ZEN (ZEN-HRP) did not consume H_2_O_2_ reduction flow [130]. Additionally, MoS_2_ can be dropped onto CNTs to increase the effective surface area for fixing the signal probe. In later research, PEI was added to the nanocomposites of MoS_2_ and MWCNT. Toluidine blue (Tb) is an alkaline biological dye, an aromatic molecule with electrochemical activity, and a signal probe with good reversibility and stability [131]. Tb was assembled on the surface of the PEI-MoS_2_-MWCNT nanocomposite to obtain signal response. Finally, AuPtNPs were introduced to fix Apt. The electrochemical aptasensor worked on the principle of the change in electrochemical signal response caused by Tb after ZEN was combined with Apt with high affinity [132].

#### 3.2.2. Graphene and Its Derivatives

Epoxy, hydroxyl, and -COOH are added to the structure of graphite when it is exposed to powerful oxidants, producing graphene oxide (GO), which is then reduced to produce reduced graphene oxide (rGO) [133].

The rGO can be functionalized with various functional groups to immobilize the antibodies. Srivastava et al. created a covalent connection between ammoniated antibody and carboxylated rGO through a strong amide bond (CO-NH). The superior sensing performance of the rGO immunosensor shows its potential application in electrochemical biosensors [134]. Shi et al. reduced GO to produce carboxyl-group-functionalized rGO by the epoxy ring opening reaction with 4-aminobenzoic acid (PABA). This rGO then reduced Au^3+^ to assemble Au-Poly (PPABA) on rGO to create hybrid nanocomposites. Ab was covalently attached to the carboxyl group, and this caused the Ab to self-assemble on the AuNP surface, increasing the amount of Ab and improving the sensor’s sensitivity to AFB1 [135]. Polypyrrole (PPy) is often combined with nanomaterials because of its great conductivity, easy preparation, and low cost. Among nanomaterials, GO/PPy nanocomposites, electrochemically reduced GO (ErGO) are suitable for biosensor applications because of their high conductivity, excellent biocompatibility, and chemical stability [136,137]. Lu et al. described a biosensor that modified the electrode surface with PPy, ErGO, and AuNPs, which significantly improved the electrochemical response and provided effective Ab immobilization. The biosensor can specifically detect the target toxin in co-existing toxins environment [138].

For the immobilization of protein, GO-modified GCE (GO/GCEs) are frequently used in electrochemical biosensors to detect various analytes [139]. Ab-BSA immunoglobulin G (IgG) of a rabbit was produced and immobilized on a GCE coated with a GO/AuNPs nanocomposite. The PAT in the sample can be captured by the Ab-BSA IgG on the biosensor surface. The reaction between IgG and PAT reduced the space blocking effect of IgG on biosensor, thereby reducing the electron transfer resistance. This electrochemical biosensor can detect PAT rapidly in less than 1 min [140].

For the immobilization of Apt, in order to better immobilize the Apt by using spacers, it is preferable to have more -COOH on GO. The initial material to be dropped onto the electrode interface was carboxyl-functionalized graphene oxide (FGO), and the Apt was then immobilized by using hexamethylenediamine (HMDA) as a spacer using carbodiimide amide-bonding chemistry. FGO can be used not only for bridging Apt but also for greatly enhancing the conductive and catalytic properties, thus amplifying the electrochemical signal of the sensor. In this sensor, the Apt labeled by MB redox probe was used as the signal fragment, and FGO was used as the signal amplification platform. Furthermore, this developed platform may be extended to other types of sensors for the detection of many other analytes [141].

#### 3.2.3. Other Carbon Nanomaterials

A metal-free semiconductor nanomaterial known as 2D graphite-like carbon nitride nanosheet (g-CNNS) was developed [142,143]. In addition to its electronic and optical properties, g-CNNS shows peroxidase-like activity. Zhu et al. created an aptasensor composed of g-CNNS and Apt. The presence of OTA causes Apt in the aptasensor to change conformation to release free signal strand DNA (ssDNA). Then g-CNNS assembles free ssDNA through π-π interactions. ssDNA acts as a mimic enzyme to catalyze the oxidation of H_2_O_2_ to generate current. Compared with earlier g-CNNS-based aptasensors, there is an excellent property that this aptasensor did not require labeled Apt and immobilization [144] (Table 7).

### 3.3. Other Nanomaterials

#### 3.3.1. Quantum Dots (QDs)

Since the oxidation potentials of metal components produce sharp and high-resolution stripping voltammetry signals, semiconductor nanocrystal quantum dots (QDs) were used for electrochemical signal transduction [145]. For signal production, PbS QD LBL assemblies can be used as signal labels. The covalent crosslinking of the external -COOH coated on the QDs with the amino groups of monoclonal antibody (MAb) was achieved for the bioconjugation of QDs with MAb. A large number of Pb^2+^ ions released by QDs during acid dissolution were detected by electrochemical detection of the concentration of metal cations released [146].

To ensure the specificity of the sensor and reduce the measurement error of mycotoxin caused by matrix effect and manual treatment, separation materials can be added in the sensor. MAb and magnetic beads are combined to create immunoaffinity magnetic beads (IMB), a new kind of separation material. Xuan et al. established a biosensor composed of IMB and CdTe QDs to detect AFB1. AFB1 was first exclusively captured by IMB, and the remaining adsorption sites were occupied by QDs labeled with Ab. Therefore, the more AFB1 in solution, the less AFB1-BSA-QDs conjugates bound with IMB. AFB1-BSA-QDs can be easily converted into corresponding metallic cations through acidic treatment, which can be detected electrochemically. Furthermore, the method was validated for its potential to detect real samples by analyzing naturally contaminated samples [147].

Silica coated with CdTe or PbS quantum dots can be used as markers to label Apt. In the presence of analyte, Apt prefers to bind to the analyte, forcing the magnetic beads (Fe_3_O_4_-Au) to partially release the preloaded label. These labels were negatively correlated with the analyte content. When specific Apt are available, it can be easily extended to simultaneously detect large amounts of mycotoxins by using different metal sulfide QDs [148].

Compared with pure QDs, ZnCdS QDs capped with ZnS are more environmentally friendly. Core shell QDs enhance electrochemiluminescence (ECL) emission to obtain stronger ECL signals. Sun et al. designed the sensor based on measuring the electrical signal generated during the redox reaction of electrogenerated reactants between electrodes and electrolyte. Nafion solution aggregates a large amount of QDs on the AuE surface as the ECL signal probe, and uses a specially coupled Ab as the capture element. The decreased ECL signals resulting from AFB1 in the samples were recorded for quantification as the reduction reaction between S_2_O_8_^2−^ in the electrolyte and QDs on the electrode led to ECL emission. Compared with the previously reported sensors, there are fewer manufacturing steps and higher sensitivity, and AFB1 in complex traditional Chinese medicine can be determined explicitly by antigen-antibody reaction [149].

CdS QDs have attracted much attention due to their ideal band gap and wide range of electrical and optical properties. They have become an excellent material for photoelectrochemistry (PEC) analysis. Based on magnetic CdS-Fe_3_O_4_ nanocomposites, Liu et al. developed a simple label free PEC platform to detect AFB1. The QDs were loaded on the surface of mesoporous Fe_3_O_4_ nanoparticles (Fe_3_O_4_NPs) via covalent bonding between -COOH and -NH_2_. anti-AFB1 was coupled to the electrode modified by CdS-Fe_3_O_4_ nanocomposites through the classical EDC coupling reaction between the carboxyl (-COOH) group on the surface of the TGA-terminated CdS QD and the amino (-NH_2_) group of the Ab. The proposed PEC biosensor is simple, sensitive, fast, and stable, opening a new promising PEC platform [150].

#### 3.3.2. Black Phosphorus and Black Phosphene (BP)

Black phosphorus is a kind of allotrope of phosphorus. There are two kinds of structures, blocky and two-dimensional single crystal structure. At present, the “dream material” under study comparable to graphene is two-dimensional single crystal black phosphorus. In a study, black phosphorus nanosheets (black phosphorusNSs) were used to modify electrode. Similar to GO, Apt can attach to the surface of black phosphorusNSs through π-π stacking between Apt and black phosphorusNSs. Thus, the electron transfer was blocked by Apt and caused high resistance. After PAT was added, the Apt specifically binds to PAT, resulting in the release of the Apt. Then, AuNPs were introduced into the sensor to compare the performance changed in the front and rear sensors. AuNPs can be connected with amine-functionalized black phosphorusNSs through Au-NH_2_. The thiolated Apt were self-assembled on nanocomposites via Au–S bonds. From the test results, the introduction of AuNPs improves the performance of the sensor [151].

Black phosphene (BP) is a 2D semiconductor material with a single atomic layer and a direct band gap composed of ordered phosphorus atoms separated from black phosphorus. Due to its low defect density, high carrier lifetime, faster hole mobility, and direct band gap, it is a graphene-like electronic nanomaterial superior to graphene. BP contains phosphorus (P) atoms, each connected with the other three P atoms, thus forming a fold structure with a large surface area [152]. Phosphonene (single layer BP) with unique functions can bridge the gap between graphene and transition metal oxide and transition metal hydride nanosheets in terms of direct band gap and high mobility. A layer-dependent bandgap from 0.3 eV to ≈2 eV, as well as high-precision optical response properties and anisotropic charge transport can be achieved by controlling the BP structure, leading to electronic and optoelectronic applications of BP. In addition, compared with other 2D nanomaterials, BP nanosheets have good biodegradability and low cytotoxicity [153]. However, BP nanosheet loses its stability under environmental conditions. It reacts highly with water and oxygen, completely degrading its electrochemical performance [154,155]. Xiang et al. prepared a sensor by using Ag^+^-functionalized BP nanosheet, which improved the stability in water, and maintained the original electrochemical performance [156]. Strong electrochemical stability, excellent electrocatalytic activity, and superior anti-fouling property are all displayed by the BP-modified electrode [157]. Based on the strategy that 2D layered BP has a large specific surface area and can load more AuNPs to improve the sensitivity of the sensor, Zhao et al. introduced nanocomposites of AuNPs and BP nanosheets into an electrochemical aptasensor. AuNPs are linked to PAT Apt and OTA cDNA via Au–S bond. Fc-functionalized OTA aptamers (Fc-OTA-Apt) and MB-functionalized PAT aptamers (MB-PAT-Apt) are used to construct the aptasensor. This technique can be used to identify PAT and OTA in apple juice and can take the role of appropriate Apt and cDNA to detect additional multiple targets [158]. Representative biosensors based on QDs, black phosphorus, and BP are listed in Table 8.

## 4. Roles of Nanomaterials in Electrochemical Biosensor for Pesticide and Mycotoxin Detection

### 4.1. Immobilization of Biomolecules

Nanomaterials are often used as carriers of biomolecules, including enzymes, Ab, Apt, cells, and tissues, due to numerous advantages. First, nanomaterials’ high specific surface area can increase the number of immobilized biomolecules. Second, nanomaterials can increase physiological stability and maintain the activity of biomolecules. Third, nanomaterials facilitate easy modification. Fourth, nanomaterials accommodate a great variety of biomolecules.

Gold, carbon-, and phosphor-based nanomaterials are frequently reported as immobilization platforms of biomolecules for biosensing. AuNPs can immobilize Ab through ionic or hydrophobic interactions. The gold surface can covalently bind to AChE through thiol bonds [159]. AChE can also bind to carboxylated MWCNT via amide bonds between the -NH_2_ group on the enzyme surface and the -COOH group on the nanotube surface. Apt can attach to the surface of black phosphorusNSs through π-π stacking between Apt and black phosphorusNSs.

Apt may cause loss of biological activity when it is immobilized to the sensor and may be unevenly distributed on the platform. The integration of nano-sized materials helps to maintain the biological activity of the Apt, while enabling these biomolecules to be evenly distributed [160]. The performance of immobilized biomolecules of the sensor can be effectively improved by surface modification [161]. Protein-modified nanomaterials can specifically bind Fc region of antibody and release antigen binding site to better ensure the biological activity of Apt.

### 4.2. Signal Generation

ECL and PEC are recently developed and constantly evolving techniques that have garnered much interest. Light is employed as the excitation source, and the resulting photocurrent is used as the detection signal in PEC analysis. Among them, QDs show great application potential [162,163]. QD is gathered on the electrode surface, and the electric signal generated by the redox reaction is transmitted to QD, causing ECL emission. In PEC detection, QD can be used as a photo active material, and the photocurrent generated decreases with the increase in analyte concentration. The analyte concentration can be determined by detecting the metal content in QD as the signal value through competitive adsorption or other methods.

### 4.3. Signal Amplification

Electrochemical analysis determines analyte contents according to the changes in the electrochemical properties of a redox probe on the electrode surface. In this way, the amount of analyte involved in the redox reaction is usually determined directly by measuring physical quantities, e.g., current and potential [164]. Nanomaterials exhibit excellent electron transfer capabilities, enhance the electrocatalytic capabilities of sensors, and can increase electron transfer between biomolecules and transducer surfaces [165,166]. Gold, carbon, phosphorus-based, and magnetic nanomaterials exhibit good electrical conductivity, facilitating rapid electron transport between electroactive molecules and electrodes for signal amplification. Meanwhile, polymer-modified nanomaterials can further enhance conductivity.

## 5. Discussion

Various electrochemical nano-biosensors are discussed in the present review; the merits and demerits of representative sensors are summarized, according to their detection roles and theories (Table 9). Among our reviewed nanomaterials, it is interesting that bimetallic nanoparticles can overcome the drawbacks of single metals in applications. Because they are fabricated from a combination of two different metals, they have the advantages of both metals and the new characteristics that arise when two metals are mixed. Therefore, bimetallic nanoparticles are becoming a popular nanomaterial for electrochemical biosensors due to their superior properties.

In addition to metal nanomaterials, 3D-DNA network aptamer sensors perform well in terms of detection sensitivity, and this modification of biomolecules improves the usability of Apt.; 3D-DNA can provide a wealth of binding sites for signal tags and signal amplifiers. However, the development of 3D-DNA network aptamer sensors is in its infancy and finding signal amplifiers with good catalytic effects on signal tags such as Thi is crucial.

## 6. Future Prospects

Considering the purpose of commercialization, the selectivity and simultaneous analysis capability of nano-biosensors need to be further investigated. Aptasensors are easy to customize, low in synthesis cost, and can be developed as point of care (POC) for medical diagnostic testing near patient care locations and times [167,168]. Biosensors are widely used in POC. Among them, screen printing technology is usually used for large-scale production of low-cost electrochemical biosensors. Moreover, the miniaturization of equipment is relatively easy, which is particularly important for the practicability of POC. However, the interference of complex sample matrix may limit the practical application of these biosensors [169].

Commercialization poses the challenge of reusability and mass production, which places greater demands on the simplicity and cost-effectiveness of some sensors. AuNPs have gained significant attention from researchers and have been developed as many excellent electrochemical biosensors. However, it is well-known that noble metals (such as gold and silver) are expensive, so such sensors need to be more practical. Improving nano-biosensor stability is also an urgent need for moving toward commercialization. Of all the sensors reviewed in this paper, the response of the most stable sensor remained remarkably constant over a storage period of 90 days, and the second stable sensor had a storage period of 60 days. Both sensors are graphene-based sensors immobilized with different biomolecules, which were antibodies and Apt, respectively. It is promising that the recently developed nano-biosensors are well-reproducible, especially those fabricated of carbon-based nanomaterials, including carbon nanotubes, graphene and their derivatives. In addition, in preparation of electrochemical nanosensors, some materials have been used along with nanoparticles to modify electrodes. However, the synthesis of modified electrodes requires much caution as some materials show toxicity and non-biocompatibility.

## 7. Conclusions

This review summarizes recent progress in determining pesticide residues and mycotoxins in food by nano-biosensors based on electrochemical methods. The methods used in the research of electrochemical biosensors in this review include current measurement, PEC, ECL, electrochemistry, and electrochemistry. The nanomaterials are discussed include noble metal nanomaterials, magnetic metal nanoparticles, metal organic framework, carbon nanotubes, graphene, and its derivatives and other materials.

In the studies summarized in this paper, for pesticide detection, the most commonly used biomaterials immobilized on the biosensors are AChE and various pesticide antibodies, while for mycotoxin detection, the most used are aptamers and antibodies. All these sensors have detection limits below national standards and excellent linear ranges. Current research on detecting pesticide and mycotoxin residues in food has focused on improving sensitivity. Future research directions include (1) immobilizing multiple biomolecules (e.g., aptamers) on the biosensors and detecting multiple analytes simultaneously, and (2) making the sensors available for mass production with excellent stability. With breakthroughs in such two aspects, the improved biosensors will facilitate better food quality control for the benefit of public health.

## Figures and Tables

**Table 1:** The main hazards of two representative categories of pesticides to human health.

Pesticides	Classification	Health Hazards	Reference
Carbamate (CB)	Insecticide	Diarrhea, respirator disorder, carcinogenic, and reproductive toxicity	[1]
Organophosphorus (OP)	Insecticide	Carcinogenic, poses potential risk to endocrine, metabolic, neurological, hepatorenal disorders, psychiatric manifestations, and neuritis.	[4]

**Table 3:** Representative studies of electrochemical biosensors for detecting pesticides from food samples by metal nanomaterials.

Nanomaterial	Sample	Analyte	Stability	Linear Range	Limit of Detection	Ref.
AuNPs/Ab	Apple, pomegranate and cabbage	OP (chlorpyrifos)	21 days (100%)	1 fM−1 μM	10 fM	[40]
AuNPs/MPS/AchE	Fruit	Carbamate	28 days (88%) 7 days (100%)	0.003–2.00 µM	1.0 nM	[41]
AuNPs/AchE	Methyl parathion	OP (Methyl parathion)	/	0.2–1 µg/L	0.6 µg/L	[42]
AuNRs/AchE	River water	OP (Paraoxon; dimethoate)	30 days (93%)	1 nM−5 µM (Paraoxon); 5 nM−1 µM (dimethoate)	0.7 nM (paraoxon); 3.9 nM (dimethoate)	[49]
AuAgNRs/AchE	River water	OP (Paraoxon; dimethoate)	/	5 nM−1 µM (Paraoxon); 10 nM−5 µM (dimethoate)	0.7 nmol/L	[49]
AgNWs/PTTBO/BchE	Tap water and milk	OP (paraoxon)	15 days (100%) 25 days (slight decrease)	10–120 µM and 0.5–8 µM	0.212 µM	[51]
Fe_3_O_4_NPs/c-MWCNT/AchE	Milk and water	OP (malathion, chlorpyrifos, monocrotophos, endosulfan)	60 days (50 uses) (75%)	0.1–40 nM (malathion); 0.1–50 nM (chlorpyrifos); 1–50 nM (monocrotophos) 10–100 nM (endosulfan)	0.1 nM (malathion and chlorpyrifos); 1 nM (monocrotophos); 10 nM (endosulfan)	[52]
Fe_3_O_4_NPs/MHCS/AchE	Practical pear	OP (malathion)	30 days (79%)	0.01–50 ppb; 50–600 ppb	0.0182 ppb	[54]
Poly(FBThF)/f-MNPs (SiO_2_-Fe_3_O_4_NPs-COOH)/AchE	Tap water	OP (Paraoxon) (Trichlorfon)	60 days (65%) 10 days (100%)	0.05–5 µg/L (paraoxon) 5–9.28 µg/L (paraoxon); 0.05–4.1 µg/L (trichlorfon) 4.1–9 µg/L (trichlorfon)	0.022 µg/L (Paraoxon); 0.037 µg/L (trichlorfon)	[55]
Cd-MOF/2-ABA/Ab	Rice	OP (parathion)	25 days (75%)	0.1–20 ng/mL	0.1 ng/mL	[59]
rGO/MoS_2_/AuNPs/AchE	Spiked vegetable water	OP (paraoxon)	7 days (96%)	0.005–0.15 μg/mL	0.0014 μg/mL	[63]
MoS_2_/AuNPs/AchE	Apple and pakchoi	OP (Paraoxon)	/	1.0–1000 μg/L	0.013 μg/L	[64]

Abbreviations: gold nanoparticles (AuNPs), antibody (Ab), organophosphorus (OP), (3-mercaptopropyl)-trimethoxy silane (MPS), acetylcholinesterase (AchE), aptamer (Apt), silver nanowires (AgNWs), 5,6-bis(octyloxy)—4,7-bis (thiopheno [3] [3,2-b] thiophene-2-yl) benzo [c] [1,2,5] oxadiazole (TTBO), butylcholinesterase (BchE), Au nanorods (AuNRs), Au–Ag heterogeneous nanorods (AuAgNRs), iron oxide nanoparticles (Fe_3_O_4_NP), carboxylated multi-walled carbon nanotubes (c-MWCNT), 4,7-bis (furan-2-yl) benzo [c] [1,2,5] thiadiazole (FBThF), functionalized magnetic nanoparticle (f-MNP), reduced graphene oxide (rGO), Cd-Metal–organic frameworks (Cd-MOFs), 2-aminobenzylamine (2-ABA).

**Table 4:** Representative studies of electrochemical biosensor for detecting pesticides from food sample by carbon-based nanomaterials.

Nanomaterial	Sample	Analyte	Stability	Linear Range	Limit of Detection	Ref.
Au/MWCNT/AChE	Paraoxon	OP (paraoxon)	/	0.1–7 nM	0.1 nM (0.025 ppb)	[66]
AuNPs/MWCNTs/c-SWCNTs/AChE/Nafion	Cabbage, onion, spinach	OP (Methyl Parathion, Monocrotophos, Chlorpyrifos and Endosulfan)	60 days	0.1–130 µM	1.9 nM (Methyl Parathion) 2.3 nM (Monocrotophos) 2.2 nM (Chlorpyrifos) 2.5 nM (Endosulfan)	[67]
VNSWCNTs/AuNPs/AChE	Cabbage water sample, tap water, purified water, river water and lake water	OP (Malathion, Methyl parathion and Chlorpyrifos)	28 days (95%) 7 days (99%)	1.00 × 10^−5^–1.00 ppb (Malathion, Methyl parathion and Chlorpyrifos)	1.96 × 10^−6^ ppb (Malathion) 3.04 × 10^−6^ ppb (Methyl parathion) 2.06 × 10^−6^ ppb (Chlorpyrifos)	[69]
CdS-G/Chitosan/AChE	OP	OP	20 days (83%)	2 ng/mL−2 μg/mL	0.7 ng/mL	[75]
GS/2-ABA/Ab	Tomato and carrot sample	OP	50 days (>95%)	0.1–1000 ng/L	52 pg/L	[76]
GQD/2-ABA/Ab	Parathion sample	OP	60 days (constant)	0.01–106 ng/L	46 pg/L	[77]

Abbreviations: poly-l-lysine (PLL), ds-DNA (double-strain DNA), multi-walled carbon nanotubes (MWCNTs), carboxylated multi-walled carbon nanotubes (c-MWCNT), vertical nitrogen-doped single-walled carbon nanotubes (VNSWCNTs), CdS-decorated graphene (CdS-G), graphene sheets (GS), 2-aminobenzylamine (2-ABA), amine-functionalized graphene quantum dots (GQD).

**Table 5:** Representative studies of aptameric biosensors for detecting pesticides from food sample.

Biosensor	Sample	Analyte	Stability	Linear Range	Limit of Detection	Ref.
6-FAM/Apt (G4Q)/ThT	Cucumber and Chinese cabbage	OP (malathion)	/	/	2.01 ppb	[79]
PANI/AuNPs/Apt	Pear juice	OP (profenofos)	/	0.1–10 µM	0.27 µM	[80]
Mo2C/Mo2N/AuNPs/Fc-CP/Apt	Apple and pakchoi	OP (chlorpyrifos)	7 days (92.3%−94.7%)	0.1–400 ng/mL	0.036 ng/mL	[81]
PDA/AuNPs/Fc-CP/Tn-Apt/Exo I	Cauliflflower and cabbage	OP (malathion)	5 days/once (four times RSD 4.48%)	0.5–650 ng/L	0.5 ng/L	[82]

Abbreviations: 6-fluorescein (6-FAM), G4-quadruplex-like (G4Q), thioflavin T (ThT), aptamer (Apt), polyaniline (PANI), gold nanoparticles (AuNPs), ferrocene-modified capture probe (Fc-CP), polydopamine (PDA), thionine (Tn), exonuclease I (Exo I).

**Table 6:** Representative studies of electrochemical biosensor for detecting mycotoxins from food sample by metal nanomaterial.

Nanomaterial	Sample	Analyte	Stability	Linear Range	Limit of Detection	Ref.
HRP/Ab/AuNPs	AFB1 solution	AFB1	12 days (90%)	0.5–10 ng/ml	0.1 ng/ml	[83]
Nafion/G/AuNPs/PhNO_2_/Ab	Cereal	DON	5 days (80.3%)	6–30 ng/mL	0.3 µg/mL	[84]
AFM1 (Apt)/AuNPs/CS	Milk and serum	AFM1	10 days (96%)	2–600 ng/L	0.9 ng/L	[85]
AuNPs/COF/Apt	Cornflour	ZEN	/	0.001–10 ng/mL	0.389 pg/mL	[86]
AuNPs–PANI/thiol-tethered-Apt/CS	Spiked maize flour	DON	/	5–30 ng/mL	3.2 ng/mL	[87]
Thi-Apt/AuNPs/CA	Grape and its commodities	OTA	/	0.1–10 ng/mL	0.03 ng/mL	[90]
CS/Apt1-Thi-AuNRs/Apt2-Fc-AuNRs	spiked beer	OTA and FB1	21 days (93.7% (Thi) and 91.4% (Fc))	0.001–100 ng/mL	0.00047 ng/mL	[93]
AgNPs/Apt	OTA	OTA	/	0.07–10 nM	0.05 nM	[94]
MOCP/Pd-PtNPs/CS/Apt/Au-PANI-Au nanohybrid	Beer	ZEN	28 days (93.5%)	1 fg/mL−100 ng/mL	0.45 fg/mL	[99]
PEI-MWCNTs/AuPtNPs/SPA-Ab	Corn flour and corn-based baby food	ZEN	10 days (89.04%)	0.005–50 ng/mL	1.5 pg/mL	[103]
Fe_3_O_4_NRs/rGO/AuNPs/CS1/Apt/hcPtAuNFs/Thi/CS2/PEI-rGO	Maize	ZEN	10 days (94.68%)	0.5 pg/mL−50 ng/mL	0.105 pg/mL	[104]
N-Cu-MOF/Apt	Spiked wheat	DON	/	0.02–20 ng/mL	0.008 ng/mL	[105]
PEI-rGO/Fe-MOF/PtAuNRs/MB-Zr-MOF/CS1/Apt/CS2	Spiked apple juice and apple wine	PAT	10 days (95.3%)	5.0 × 10^−5–^5.0 × 10^−1^ ng/mL	4.14 × 10^−5^ ng/mL	[108]
CoSe_2_/AuNRs/3dsDNA-PtNi/Co-MOF/Apt	Maize	ZEN	21 days (93.1%)	10.0 fg/mL−10.0 ng/mL	1.37 fg/mL	[110]
rMoS_2_/AuNPs/CS1/Apt1/Thi MoS_2_/AuNPs/CS2/Apt2/FC6S	Maize	ZEN, FB1	14 days (90.2% (FB1)) 14 days (90.0% (ZEN))	0.001–10 ng/mL (ZEN); 0.001–100 ng/mL (FB1)	0.0005 ng/mL	[112]
ZnONRs/chitosan/Thi-AuNPs/Apt	Spiked juice	PAT	7 days (94.4%)	50 ng/mL−0.5 pg/mL	0.27 ng/mL	[116]

Abbreviations: horseradish peroxidase (HRP), gold nanoparticles (AuNPs), antibody (Ab), graphene (G), 4-nitrophenyl (PhNO_2_), aptamer (Apt), complementary strand of the Apt (CS), covalent organic frameworks (COFs), polymer polyaniline (PANI), cysteamine (CA), thionine (Thi), thiolated ferrocene (Fc), Ag nanoparticles (AgNPs), metal organic coordination polymers (MOCPs), palladium nanoparticles and platinum nanoparticles (Pd-PtNPs), staphylococcal protein A (SPA), polyethylenimine (PEI), gold-platinum alloy nanoparticles (AuPtNPs), Fe_3_O_4_ nanorods (Fe_3_O_4_NRs), hollow cubic gold-platinum nanoframes (hcPtAuNFs), nitrogen-doped copper metal organic framework (N-Cu-MOF), polyethyleneimine-reduced graphene oxide (PEI-rGO), iron-based metal organic framework (Fe-MOF), Zr-Metal–organic frameworks (Zr-MOFs), methylene blue (MB), Co-Metal–organic frameworks (Co-MOFs), 3D structure DNA (3dsDNA), PtNi nanoclusters (PtNi), 2-aminobenzylamine (2-ABA), Zinc oxide nanorods (ZnONRs).

**Table 7:** Representative studies of electrochemical biosensor for detecting mycotoxins from food sample by carbon-based nanomaterials.

Nanomaterial	Sample	Analyte	Stability	Linear Range	Limit of Detection	Ref.
MWCNTs/sol-gel/AFO	AFB1 solution	AFB1	23 days (97.1%) 7 days (99.4%)	3.2–721 nmol/L	1.6 nmol/L	[119]
c-MWCNTs/Ab/BSA	AFB1 solution	AFB1	45 days (92%)	0.25–1.375 ng/ml	0.08 ng/ml	[120]
MWCNTs/RTIL/Ab	Olive oil	AFB1	60 days (87%)	0.1–10 ng/mL	0.03 ng/mL	[123]
PDMA-MWCNT/Ab	Certified Corn Reference Material	FB1	5 days (81%)	7–49 ng/L	3.8 pg/L	[124]
SWCNT/chitosan/FB1-BSA/Ab	Spiked corn	FB1	5 days (60%)	0.01–1000 ng/mL	2 pg/mL	[127]
CS/Apt/SWCNT/MB	Serum and grape juice	OTA	/	134–58 pM	52 pM	[128]
PEI-MWCNTs/AuNPs/Apt	Maize	ZEN	5 days (88%)	0.0001–0.1 ng/mL	0.15 pg/mL	[130]
PEI-MoS_2_/MWCNTs/Tb/PtAuNPs/Apt	Beer	ZEN	25 days (87.2%) 15 days (95.3%)	0.5 pg/mL−50 ng/mL	0.17 pg/mL	[132]
BSA/anti-AFB1/rGO	AFB1 solution	AFB1	45 days (no signicant decrease)	0.125–1.5 ng/ml	0.15 ng/ml	[134]
Au-Poly (PPABA)/rGO/Ab	vegetable oil	AFB1	10 days (96.3%)	0.01–25 ng/mL	0.001 ng/mL	[135]
ErGO-PPy/AuNPs/Ab	Spiked corn	DON	12 days (96.6%)	0.05–1 ppm (DON) 0.2–4.5 ppm (FB1)	8.6 ppb (DON) 4.2 ppb (FB1)	[138]
GO/AuNPs/IgG	PAT	PAT	/	5–200 µg/L	5 µg/L	[140]
FGO/HMDA/Apt	Alcoholic beverage	AFB1	/	0.05 ng/mL	0.05–6.0 ng/mL	[141]
g-CNNS/Apt	Red wines, juices, and corn	OTA	/	0.2–500 nM	0.073 nM	[144]

Abbreviations: aflatoxin oxidase (AFO), multi-walled carbon nanotubes (MWCNTs), carboxylated multi-walled carbon nanotubes (c-MWCNT), poly (2,5-dimethoxyaniline) (PDMA), bovine serum albumin (BSA), ionic liquids (ILs), poly (2,5-dimethoxyaniline) (PDMA), single wall carbon nanotubes (SWCNT), toluidine blue (Tb), polyethylenimine (PEI), gold-platinum alloy nanoparticles (AuPtNPs), reduced graphene oxide (rGO), 4-aminobenzoic acid (PABA), polypyrrole (PPy), electrochemically reduced GO (ErGO), immunoglobulin G (IgG), carboxyl-functionalized graphene oxide (FGO), hexamethylenediamine (HMDA), graphite-like carbon nitride nanosheet (g-CNNS).

**Table 8:** Representative studies of electrochemical biosensors for detecting mycotoxins from food samples by other nanomaterials.

Nanomaterial	Sample	Analyte	Stability	Linear Range	Limit of Detection	Ref.
QDs (PbS)/mAb	Real peanut sample	AFB1	/	0.04–15 ng/mL	0.018 ng/mL	[146]
BSA/mAb/IMB/QDs (CdTe)	Wheat, maize, husky rice, and peanut oil	AFB1	/	0.08–800μg/kg	0.05μg/kg	[147]
MBs (Fe_4_-Au)/CS/Apt/QDs (CdTe)/SiO_2_ and MBs (Fe_3_O_4_-Au)/CS/Apt/QDs (PbS)/SiO_2_	Maize sample	FB1; OTA	21 days (98.7% Cd2+; 98.1% Pb2+)	0.05–50 ng/mL (FB1) 0.01–10 ng/mL (OTA)	20 pg/mL (FB1) 5 pg/mL (OTA)	[148]
QDs (ZnCdS/ZnS)/Au/Nafion composite film/Ab	Lotus seed	AFB1	/	0.05–100 ng/mL	0.01 ng/mL	[149]
QDs (CdS)/Fe_3_O_4_/Ab	Corn sample	AFB1	12 days (91%)	0.01–80 ng/mL	5.0 pg/mL	[150]
Black phosphorus NSs/Apt	Spiked apple juice sample	PAT	/	1 nM−1µM	0.3 nM	[151]
Black phosphorus NSs/AuNPs/thiolated Apt	Spiked apple juice sample	PAT	/	0.1 nM−10.0µM	0.03 nM	[151]
Ag^+^-BP	Alcoholic beverage samples	AFB1	/	0.05 ng/mL	0.05–6.0 ng/mL	[157]
AuNPs/BP/Fc-Apt (OTA)/Mb-Apt (PAT)	Apple juice	OTA, PAT	21 days (92.0%)	0.01 × 10^−7^ µg/mL −0.10 µg/mL	/	[158]

Abbreviations: quantum dots (QDs), monoclonal antibodies (mAbs), immunoaffinity magnetic beads (IMB), magnetic beads (MB), mesoporous Fe_3_O_4_ nanoparticles (Fe_3_O_4_NP), black phosphorus nanosheets (black phosphorusNSs), black phosphene (BP), methylene blue-functionalized PAT aptamers (Mb-PAT-aptamers), ferrocene-functionalized OTA aptamers (Fc-OTA-aptamers).

**Table 9:** Merits and demerits of major nanomaterial-based electrochemical biosensors for detecting pesticides and mycotoxins.

Nanomaterial	Merits	Demerits
Au nanomaterial	Easily decorated to increase the binding area, good electrical conductivity, easy to immobilize biomolecules.	Specific surface area is relatively small, poor detection stability of the sensor, easy to form irreversible aggregation, seriously affect by the environment.
Ag nanomaterial	Among all metals, has the highest electrical conductivity, among all metals, has the best thermal conductivity, among all metals, has the best reflectivity, almost completely harmless to the human body.	Specific surface area is relatively small, for AChE immobilization, has worse catalytic ability than Au, the stability of the sensor is low.
CNTs	High surface area, abundant reaction sites, excellent electrochemical stability, high thermal conductivity, good mechanical and chemical stability, the sensor has good repeatability.	Poor dispersion, poor biocompatibility.
G/GO/rGO/ErGO	Abundant reaction sites, higher surface area than CNT, better conductivity and thermal conductivity than CNT, rGO has better conductivity, better dispersion than GO, easy manufacturing and relatively low cost than GO, sensor has good stability and repeatability.	Expensive and difficult to produce on a large scale, G is unstable with oxygen and heat, large graphene sheets contain some toxicity and impurities, size and thickness of G sheets are difficult to control.
Bimetallic nanomaterials	Combine the advantages of two metal elements.	Poor detection stability of the sensor, contains the disadvantages of two metal elements.
MNPs	Superparamagnetic or ferromagnetic, large surface area, high charge transfer capacity, excellent renewability.	High reactivity, low stability, potential genotoxicity.
MOF	Good structural tenability, high surface area,	Poor electronic conductivity, poor water stability.
QDs	Unique photocatalytic properties, long fluorescence lifetime.	High biological toxicity, chemical properties are relatively unstable, high demand for synthesis conditions, poor water solubility.
Black phosphorus and black phosphene BP	Good biodegradability, low cytotoxicity.	Low stability, seriously affect by the environment, reacts highly with water and oxygen, little research in the field of electrochemical biosensors.

## Data Availability

Not applicable.

## Abbreviations

**Abbreviations**	** Full Form**	**Abbreviations**	** Full Form**
Ab	antibody	hcPtAuNFs	hollow cubic gold-platinum nanoframes
AChE	acetylcholinesterase	HMDA	hexamethylenediamine
AF	aflatoxin	HRP	horseradish peroxidase enzyme
AFO	aflatoxin oxidase	IgG	immunoglobulin G
AgNMs	sliver nanomaterials	ILs	ionic liquids
AgNPs	Ag nanoparticles	IMB	immunoaffinity magnetic beads
AgNRs	Ag nanorods	ITO	indium tin oxide
AgNWs	Ag nanowires	JECFA	Joint Expert Committee on Food Additives
a-NP	a-naphthyl phosphate	LBL	Layer-by-layer
ATCI	acetylthiocholine iodide	LC-FLD	liquid chromatography coupled with fluorescence detection
AuAgNRs	Au–Ag heterogeneous nanorods	MAb	monoclonal antibody
AuE	gold electrode	MB	methylene blue
AuNPs	gold nanoparticle	MHCS	mesoporous hollow carbon spheres
AuNPs/G/PhNO_2_	AuNPs-dotted 4-nitrophenylazo-functionalised graphene	MNPs	Magnetic nanoparticles
AuNRs	gold nanorods	MOCPs	metal organic coordination polymers
AuPtNPs	gold-platinum alloy nanoparticles	MOF	Metal–organic framework
BChE	butylcholinesterase	MoS_2_	Molybdenum disulfide
black phosphorusNSs	black phosphorus nanosheets	MPS	(3-mercaptopropyl)-trimethoxysilane
BP	black phosphene	MS	mass spectrometry detection
BSA	bovine serum albumin	Nb. BbvCI	nicking endonuclease
CA	cysteamine	N-Cu-MOF	nitrogen-doped copper metal–organic backbone
CB	carbamate	NH_2_	amino groups
CdS-G	CdS-decorated graphene	Ni	nickel
CFME	carbon fiber microelectrodes	Ni-MOF	nickel-based metal–organic framework
c-MWCNT	carboxylated multi-walled carbon nanotubes	NMOF	nano-MOF
CNTs	carbon nanotubes	OP	organophosphorus
Co	cobalt	OTA	ochratoxin A
COFs	covalent organic frameworks	P	phosphorus
Co-MOF	cobalt-based metal–organic framework	PABA	4-aminobenzoic acid
COOH	carboxyl groups	PANI	polymer polyaniline
CS	complementary strand	PAT	patulin
Cu-MOF	copper-based metal–organic framework	PDMA	poly (2,5-dimethoxyaniline)
DAD	diode-array detection	PdNPs	Palladium nanoparticles
DON	deoxynivalenol	PEC	photoelectrochemistry
DPV	differential pulse voltammetry	PEI	polyethylenimine
ECL	electrochemiluminescence	PI	polyimide
EIS	electrochemical impedance spectroscopy	PtNi	PtNi nanoclusters
ErGO	electrochemically reduced GO	PtNPs	platinum nanoparticles
FAO	Food and Agriculture Organization	QDs	quantum dots
FBThF	4,7-bis (furan−2-yl) benzo [c] [1,2,5] thiadiazole	rGO	reduced graphene oxide
Fc	ferrocene	rMoS_2_	reduced MoS_2_
FC6S	6-(Ferrocenyl) hexanethiol	SPA	Staphylococcal protein A
Fe	iron	SPCE	screen printed carbon electrodes
Fe_3_O_4_	iron oxides	ssDNA	signal strand DNA
Fe_3_O_4_NP	iron oxide nanoparticles	SWCNT	single-walled carbon nanotubes
Fe_3_O_4_NRs	Fe_3_O_4_ nanorods	Tb	Toluidine blue
Fe-MOF	iron-based metal–organic framework	Thi	thionine
FGO	Carboxyl-functionalized graphene oxide	TLC	thin-layer chromatography
FM	fumonisin	TMDs	transition metal dichalcogenides
f-MNP	functionalized magnetic nanoparticle	TTBO	5,6-bis(octyloxy)-4,7-bis (thiopheno [3] [3,2-b] thiophene-2-yl) benzo [c] [1,2,5] oxadiazole
FTO	fluorine-doped tin-oxide electrode	VNSWCNTs	vertical nitrogen-doped single-walled carbon nanotubes
GA	Glutaraldehyde	WHO	World Health Organization
GC	gas chromatography	ZEN	zearalenone
g-CNNS	2D graphite-like carbon nitride nanosheet	ZnONRs	Zinc oxide nanorods
GO	graphene oxide	Zr-MOF	zirconium-based metal–organic framework
GQD	graphene quantum dots	2-ABA	2-aminobenzylamine
GS	graphene sheets	2D	two-dimensional
